# Association Analysis in Children Born from Normal and Complicated Pregnancies—Cardiovascular Disease Associated microRNAs and the Incidence of Prehypertension/Hypertension, Overweight/Obesity, Valve Problems and Heart Defects

**DOI:** 10.3390/ijms21218413

**Published:** 2020-11-09

**Authors:** Ilona Hromadnikova, Katerina Kotlabova, Ladislav Krofta, Jan Sirc

**Affiliations:** 1 Department of Molecular Biology and Cell Pathology, Third Faculty of Medicine, Charles University, 100 00 Prague, Czech Republic; katerina.kotlabova@lf3.cuni.cz; 2 Institute for the Care of the Mother and Child, Third Faculty of Medicine, Charles University, 147 00 Prague, Czech Republic; ladislav.krofta@upmd.eu (L.K.); jan.sirc@upmd.eu (J.S.)

**Keywords:** body mass index, cardiovascular risk, children, echocardiography, heart defects, microRNA, pregnancy-related complications, prehypertension/hypertension, overweight/obesity, valve problems

## Abstract

The goal was to assess how a history of any kind of pregnancy-related complication altered expression profile of microRNAs played a role in the pathogenesis of diabetes, cardiovascular and cerebrovascular diseases in the peripheral blood leukocytes of children at the age of 3–11 years. The prior exposure to gestational hypertension, preeclampsia, fetal growth restriction, gestational diabetes mellitus, preterm prelabor rupture of membranes or spontaneous preterm birth causes that a significant proportion of children (57.42% to 90.0% specifically) had a substantially altered microRNA expression profile, which might be the origin of a lifelong cardiovascular risk. A total of 23 out of 29 tested microRNAs were upregulated in children born from such complicated gestation. The occurrence of overweight, obesity, valve problems and heart defects even intensified upregulation of microRNAs already present in children exposed to such pregnancy complications. The occurrence of overweight/obesity (miR-92a-3p, and miR-210-3p) and valve problems or heart defects (miR-342-3p) induced microRNA upregulation in children affected with pregnancy complications. Overall, 42.86% overweight/obese children and 27.36% children with valve problems or heart defects had even higher microRNA levels than children with normal clinical findings after complicated pregnancies. In addition, the microRNA expression profile was also able to differentiate between children descending from normal gestation in relation to the occurrence of overweight and obesity. Screening on the base of the combination of 19 microRNAs identified 70.0% overweight/obese children at 90.0% specificity. In general, children after complicated pregnancies, just as children after normal pregnancies, with abnormal findings are at a higher risk of the onset of cardiovascular complications, and their dispensarization, with the aim to implement primary prevention strategies, would be beneficial.

## 1. Introduction

The increasing worldwide prevalence of pregnancy-related complications generates an increasing number of children with a lifelong cardiovascular risk [1,2,3,4,5,6,7,8,9,10,11,12,13,14,15,16,17,18,19,20,21,22,23,24,25,26,27,28,29,30,31,32,33,34,35,36,37,38,39,40,41,42,43,44,45,46,47,48,49,50,51].

Lifelong cardiovascular risk of children descending from pregnancy-related complications is based on the presence of overweight/obesity (increased body mass index and/or wider waist circumference) [1,2,3,4,5,6,7,8,9,10,11], higher systolic and/or diastolic blood pressures [1,2,6,7,8,12,13,14,15,16,17,18,19,20,21,22], higher nocturnal systolic and diastolic blood pressures [16], altered lipid profiles [6,21], altered metabolite pattern [23], impaired glucose tolerance [3,5,6,7,24], alteration in the systemic and the pulmonary circulation [25], altered cardiac geometry and function [26,27,28,29] and the acceleration of pubertal timing [30].

Some children born from complicated pregnancies have already been suffering from severe diseases, such as asthma [31,32], pulmonary hypertension [33,34,35], diabetes mellitus [36], cardiorenal metabolic syndrome [37], chronic kidney diseases [38,39], cardiovascular diseases [40], ophthalmic disorders [41], neurodevelopmental and neuropsychiatric disorders [42,43,44,45,46,47,48,49,50,51], most likely as a result of abnormal fetal programming.

Recently, we reported that a proportion of children affected with gestational hypertension (GH), preeclampsia (PE), fetal growth restriction (FGR) and gestational diabetes mellitus (GDM) had altered microRNA expression profiles that may contribute to the predisposition of these children to later onset of diabetes mellitus, cardiovascular and cerebrovascular diseases [52,53].

Prenatal exposure to maternal complications (GH, PE and GDM) or fetal complication (FGR) was associated in a proportion of children with deregulation of miR-1–3p, miR-17-5p, miR-20a-5p, miR-20b-5p, miR-21-5p, miR-23a-3p, miR-26a-5p, miR-29a-3p, miR-103a-3p, miR-125b-5p, miR-126-3p, miR-133a-3p, miR-146a-5p, miR-181a-5p, miR-195-5p, miR-210-3p and miR-342-3p [52,53].

In addition, a prior exposure to GDM induced postnatal deregulation of miR-16-5p, miR-92a-3p, miR-100-5p, miR-143-3p, miR-155-5p, miR-221-3p, miR-499a-5p and miR-574-3p [53].

The pivotal goal of the current study was to assess how a history of any kind of pregnancy complication (GH, PE, FGR, GDM, preterm prelabor rupture of membranes or spontaneous preterm birth) might contribute to postnatal alterations of microRNA expression profiles in whole peripheral venous blood (leukocytes).

In addition, the subject of our interest was extended to explore the impact of the actual occurrence of individual abnormal findings (overweight, obesity, prehypertension, hypertension, valve problems and heart defects) on already present abnormal microRNA expression profiles in children born from complicated gestation.

An additional goal of the current study was to determine to what extent individual abnormal findings (overweight, obesity, prehypertension, hypertension, valve problems and heart defects) might influence microRNA expression profiles in children born from normal gestation.

Any data on the association between microRNA expression profiles (miR-1-3p, miR-16-5p, miR-17-5p, miR-20a-5p, miR-20b-5p, miR-21-5p, miR-23a-3p, miR-24-3p, miR-26a-5p, miR-29a-3p, miR-92a-3p, miR-100-5p, miR-103a-3p, miR-125b-5p, miR-126-3p, miR-130b-3p, miR-133a-3p, miR-143-3p, miR-145-5p, miR-146a-5p, miR-155-5p, miR-181a-5p, miR-195-5p, miR-199a-5p, miR-210-3p, miR-221-3p, miR-342-3p, miR-499a-5p and miR-574-3p) in peripheral white blood cells, and the occurrence of overweight/obesity, prehypertension/hypertension, valve problems or heart defects in children aged 3–11 years born from normal and complicated gestation are currently not available.

The idea of the determination of potential diabetic/cardiovascular risk in children prenatally or perinatally affected by any kind of pregnancy-related complication was based on the fact that appropriate microRNAs are involved in the inducement and progress of diabetes mellitus, cardiovascular and cerebrovascular diseases (Table S1). We selected 29 microRNAs, which had usually been displaying aberrant expression in plasma/serum/affected tissue samples of adult and elderly patients with signs of diabetes mellitus, cardiovascular and cerebrovascular diseases.

## 2. Results

### 2.1. Abnormal microRNA Expression Profile in Children Born from Complicated Pregnancies

At first, we made a comparison of microRNA expression profile in children born from normal and complicated gestation, irrespective of the type of pregnancy-related complication (gestational diabetes mellitus, gestational hypertension, preeclampsia, fetal growth restriction, preterm prelabor rupture of membranes and/or spontaneous preterm birth). Both the Mann–Whitney (M-W) and receivers operating characteristic (ROC) curve analyses revealed increased expression of 23 out of 29 tested microRNAs (miR-1-3p, miR-16-5p, miR-17-5p, miR-20a-5p, miR-20b-5p, miR-21-5p, miR-23a-3p, miR-26a-5p, miR-29a-3p, miR-100-5p, miR-103a-3p, miR-125b-5p, miR-126-3p, miR-130b-3p, miR-133a-3p, miR-143-3p, miR-146a-5p, miR-181a-5p, miR-195-5p, miR-199a-5p, miR-221-3p, miR-499a-5p and miR-574-3p) in children born from complicated gestation. MicroRNAs with aberrant expression profile displayed a sensitivity with a range 10.71–31.39% at a 10.0% false positive rate (FPR) (Figure S1).

**Figure 1:**
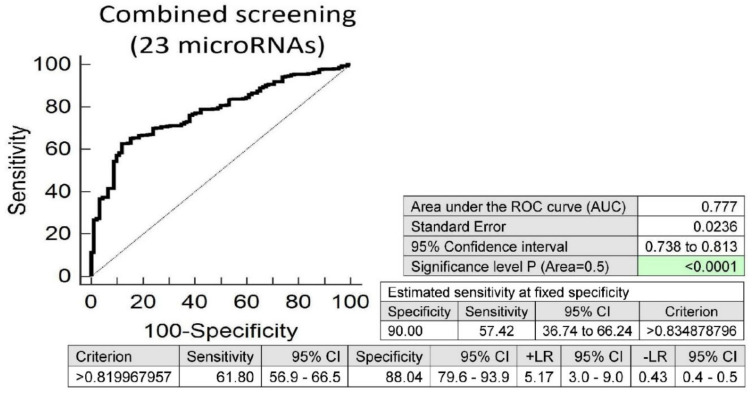
Aberrant microRNA expression profile in children descending from complicated pregnancies, irrespective of the type of pregnancy-related complication (gestational diabetes mellitus, gestational hypertension, preeclampsia, fetal growth restriction, preterm prelabor rupture of membranes and/or spontaneous preterm birth). Combined screening revealed that at a 10.0% false positive rate (FPR) 57.42% children prenatally exposed to any pregnancy-related complication had substantially altered microRNA expression profile (miR-1-3p, miR-16-5p, miR-17-5p, miR-20a-5p, miR-20b-5p, miR-21-5p, miR-23a-3p, miR-26a-5p, miR-29a-3p, miR-100-5p, miR-103a-3p, miR-125b-5p, miR-126-3p, miR-130b-3p, miR-133a-3p, miR-143-3p, miR-146a-5p, miR-181a-5p, miR-195-5p, miR-199a-5p, miR-221-3p, miR-499a-5p and miR-574-3p).

Screening on the base of a combination of all above mentioned microRNAs showed that, at 10.0% FPR, 57.42% of children born from complicated gestation had significantly altered postnatal microRNA expression profile, which may indicate an increased risk of later onset of diabetes mellitus, cardiovascular and cerebrovascular diseases (area under the curve (AUC) 0.777, *p* < 0.001, sensitivity 61.80%, specificity 88.04%, cut off >0.819967957) (Figure 1).

Subsequently, we performed comparison of microRNA expression profile between particular groups of children with respect to actual findings (normal systolic and diastolic blood pressures vs. prehypertension/hypertension; normal body mass index (BMI) vs. overweight/obesity; normal values of echocardiographic measurements vs. the occurrence of valve problems or heart defects), regardless of prenatal or perinatal exposure to pregnancy complications (gestational diabetes mellitus, gestational hypertension, preeclampsia, FGR, preterm prelabor rupture of membranes and/or spontaneous preterm birth).

### 2.2. No Association between the Presence of Prehypertension/Hypertension and Expression of Examined microRNAs

Overall, 63 out of 503 (12.53%) tested children were confirmed over several visits to have systolic prehypertension/hypertension, and 58 out of 503 (11.53%) tested children had diastolic prehypertension/hypertension.

No association between the occurrence of prehypertension or hypertension (combined systolic and diastolic prehypertension/hypertension and/or isolated systolic or diastolic prehypertension/hypertension defined as systolic and/or diastolic blood pressures equal or above the 90th or 95th percentiles) and the expression of examined microRNAs was identified in children, regardless of the course of gestation of their mothers.

### 2.3. Postnatal Expression Profile of 19 Tested microRNAs Differentiates between Overweight/Obese Children and Children with Nomal BMI Values

Overall, 32 out of 503 (6.36%) tested children were confirmed to be overweight/obese.

A total of 19 out of 29 tested microRNAs (miR-1-3p, miR-16-5p, miR-17-5p, miR-21-5p, miR-23a-3p, miR-24-3p, miR-26a-5p, miR-92a-3p, miR-100-5p, miR-103a-3p, miR-125b-5p, miR-126-3p, miR-130b-3p, miR-133a-3p, miR-146a-5p, miR-181a-5p, miR-210-3p, miR-221-3p and miR-574-3p) showed increased expression in overweight/obese children (defined as BMI equal to or above the 85th or 95th percentiles) when the comparison to children with normal BMI values regardless of prenatal and perinatal outcomes was performed. The ROC curve analyses showed that these particular microRNAs differentiated at 10.0% FPR between overweight/obese children and children with normal BMI values, with a sensitivity ranging from 6.25% to 31.25% (Figure S2).

**Figure 2:**
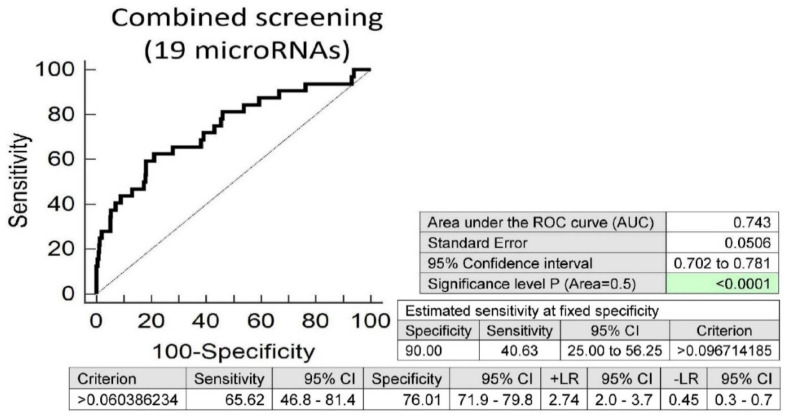
Aberrant microRNA expression profile in overweight/obese children, irrespective of the course of gestation of their mothers (normal and complicated pregnancies altogether). Combined screening revealed that at 10.0% FPR, 40.63% of overweight/obese children had substantially altered microRNA expression profile (miR-1-3p, miR-16-5p, miR-17-5p, miR-21-5p, miR-23a-3p, miR-24-3p, miR-26a-5p, miR-92a-3p, miR-100-5p, miR-103a-3p, miR-125b-5p, miR-126-3p, miR-130b-3p, miR-133a-3p, miR-146a-5p, miR-181a-5p, miR-210-3p, miR-221-3p and miR-574-3p).

Screening on the base of a combination of these 19 aberrantly expressed microRNAs was superior to single microRNAs. At 10.0% FPR, 40.63% overweight/obese children had substantially altered microRNA expression profile, which may contribute to the predisposition to an increased risk of later onset of diabetes mellitus, cardiovascular and cerebrovascular diseases (AUC 0.743, *p* < 0.001, sensitivity 65.62%, specificity 76.01%, cut off >0.060386234) (Figure 2).

### 2.4. Postnatal Expression Profile of 10 Tested microRNAs Differentiates between Children with Abnormal and Normal Values of Echocardiographic Measurements

Overall, 123 out of 503 (24.45%) tested children were confirmed to have abnormal values of echocardiographic measurements. In detail, children indicated by the sonographer during the visit had the following valve problems and heart defects ((tricuspid valve regurgitation (*n* = 57/503), mitral valve regurgitation (*n* = 5/503), pulmonary valve regurgitation (*n* = 22/503), bicuspid aortic valve regurgitation (*n* = 2/503), ventricular septum defect (*n* = 4/503), atrial septum defect (*n* = 3/503), foramen ovale apertum (*n* = 40/503), ductus arteriosus patens (*n* = 3/503), and arrhythmia (*n* = 3/503)).

A total of 10 out of 29 tested microRNAs (miR-1-3p, miR-16-5p, miR-20a-5p, miR-21-5p, miR-125b-5p, miR-126-3p, miR-146a-5p, miR-195-5p, miR-221-3p and miR-499a-5p) showed increased expression in children with valve problems or heart defects when compared to children with normal echocardiographic values, regardless of prenatal and perinatal outcomes. The ROC curve analyses showed that these individual microRNAs differentiated at 10.0% FPR between children with normal and abnormal values of echocardiographic measurements, with a sensitivity ranging from 10.66% to 19.67% (Figure S3).

**Figure 3:**
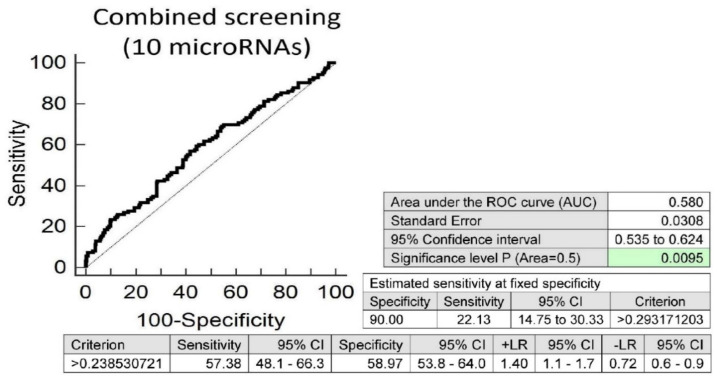
Aberrant microRNA expression profile in children with valve problems or heart defects, irrespective of the course of gestation of their mothers (normal and complicated pregnancies altogether). Combined screening revealed that at 10.0% FPR, 22.13% of children with valve problems or heart defects had substantially altered microRNA expression profile (miR-1-3p, miR-16-5p, miR-20a-5p, miR-21-5p, miR-125b-5p, miR-126-3p, miR-146a-5p, miR-195-5p, miR-221-3p and miR-499a-5p).

Screening on the base of a combination of these 10 aberrantly expressed microRNAs showed a slightly higher accuracy than individual microRNAs, with the best performance (miR-21-5p, miR-499a-5p and miR-126-3p). It showed that at 10.0% FPR, 22.13% of children with valve problems or heart defects had a substantially altered microRNA expression profile (AUC 0.580, *p* = 0.0095, sensitivity 57.38%, specificity 58.97%, cut off >0.238530721) (Figure 3).

Nevertheless, with regard to a high number of outliers in children with normal BMI values and in children with normal values of echocardiographic measurements, and with regard to a large impact of the course of gestation of mothers on postnatal microRNA expression profile, we finally decided to also evaluate microRNA expression data within the equal groups of children (children born from normal gestation only, and children born from complicated gestation only).

### 2.5. Postnatal Expression Profile of 19 Tested microRNAs Differentiates between Overweight/Obese and Normal Weight Children Born from Normal Pregnancies

Overall, 10 out of 92 (10.87%) tested children born from normal gestation were confirmed to be overweight/obese.

In children born from normal gestation, the expression of microRNAs was significantly different or on a border of significance (miR-16-5p, miR-17-5p, miR-20b-5p, miR-21-5p, miR-23a-3p, miR-24-3p, miR-26a-5p, miR-29a-3p, miR-92a-3p, miR-100-5p, miR-103a-3p, miR-125b-5p, miR-126-3p, miR-130b-3p, miR-145-5p, miR-146a-5p, miR-181a-5p, miR-210-3p and miR-574-3p) in overweight/obese individuals. The ROC curve analyses showed that these individual microRNAs differentiated at 10.0% FPR between overweight/obese and normal weight children, with a sensitivity ranging from 10.0% to 40.0% (Figure S4).

**Figure 4:**
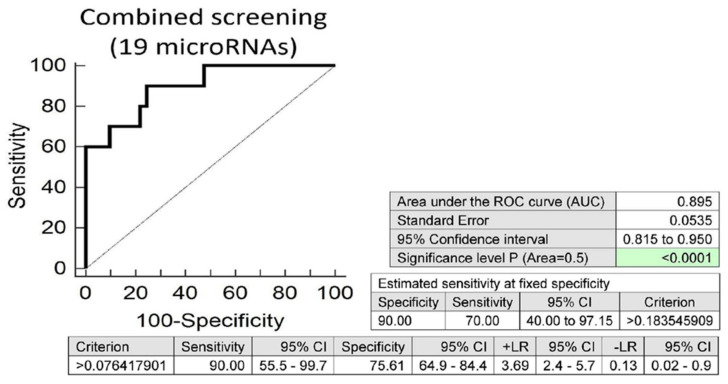
Aberrant microRNA expression profile in overweight/obese children descending from normal pregnancies. Combined screening revealed that at 10.0% FPR, 70.0% of overweight/obese children had a substantially altered microRNA expression profile (miR-16-5p, miR-17-5p, miR-20b-5p, miR-21-5p, miR-23a-3p, miR-24-3p, miR-26a-5p, miR-29a-3p, miR-92a-3p, miR-100-5p, miR-103a-3p, miR-125b-5p, miR-126-3p, miR-130b-3p, miR-145-5p, miR-146a-5p, miR-181a-5p, miR-210-3p and miR-574-3p).

Screening on the base of a combination of all these 19 microRNAs was superior to the performance of single microRNAs, and showed that at 10.0% FPR, 70.0% of overweight/obese children born from normal gestation had a substantially altered postnatal microRNA expression profile (AUC 0.895, *p* < 0.001, sensitivity 90.0%, specificity 75.6%, cut off >0.076417901) (Figure 4).

### 2.6. Postnatal Expression Profile of 18 Tested microRNAs Differentiates between Overweight/Obese and Normal Weight Children Born from Complicated Gestation

Overall, 22 out of 411 (5.35%) tested children born from complicated gestation were confirmed to be overweight/obese.

In children born from complicated gestation, increased expression or a trend towards increased expression (miR-1-3p, miR-16-5p, miR-17-5p, miR-20a-5p, miR-26a-5p, miR-92a-3p, miR-103a-3p, miR-125b-5p, miR-126-3p, miR-130b-3p, miR-133a-3p, miR-146a-5p, miR-181a-5p, miR-195-5p, miR-199a-5p, miR-210-3p, miR-221-3p and miR-499a-5p) was detected in overweight/obese children. The ROC curve analyses showed that these individual microRNAs differentiated at 10.0% FPR between overweight/obese and normal weight children, with a sensitivity ranging from 9.09% to 31.82% (Figure S5).

**Figure 5:**
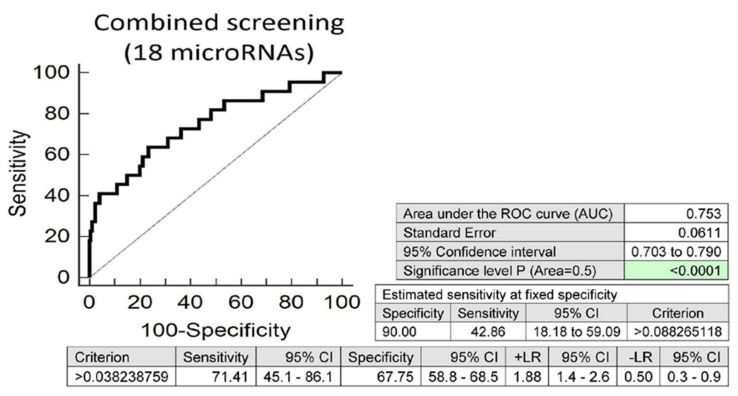
Aberrant microRNA expression profile in overweight/obese children descending from complicated pregnancies. Combined screening revealed that at 10.0% FPR, 42.86% of overweight/obese children had a substantially altered microRNA expression profile (miR-1-3p, miR-16-5p, miR-17-5p, miR-20a-5p, miR-26a-5p, miR-92a-3p, miR-103a-3p, miR-125b-5p, miR-126-3p, miR-130b-3p, miR-133a-3p, miR-146a-5p, miR-181a-5p, miR-195-5p, miR-199a-5p, miR-210-3p, miR-221-3p and miR-499a-5p).

Screening on the base of a combination of all these 18 microRNAs was superior to the performance of single microRNAs, and showed that at 10.0% FPR, 42.86% of overweight/obese children born from complicated gestation had a significantly altered postnatal microRNA expression profile (AUC 0.753, *p* < 0.001, sensitivity 71.4%, specificity 67.7%, cut off >0.038238759) (Figure 5).

### 2.7. Postnatal Expression Profile of 19 microRNAs Differentiates between Children with Abnormal and Normal Values of Echocardiographic Measurements Descending from Complicated Pregnancies

Overall, 106 out of 411 (25.79%) tested children born from complicated gestation had abnormal values of echocardiographic measurements. In detail, children indicated by the sonographer during the visit had the following valve problems and heart defects ((tricuspid valve regurgitation (*n* = 49/411), mitral valve regurgitation (*n* = 4/411), pulmonary valve regurgitation (*n* = 20/411), bicuspid aortic valve regurgitation (*n* = 1/411), ventricular septum defect (*n* = 3/411), atrial septum defect (*n* = 2/411), foramen ovale apertum (*n* = 35/411), ductus arteriosus patens (*n* = 3/411) and arrhythmia (*n* = 2/411)).

In children born from complicated gestation, the statistical significant difference or difference on a border of significance was detected between individuals with abnormal and normal values of echocardiographic measurements for miR-1-3p, miR-16-5p, miR-17-5p, miR-20a-5p, mir-20b-5p, miR-21-5p, miR-26a-5p, miR-29a-3p, miR-100-5p, miR-125b-5p, miR-126-3p, miR-143-3p, miR-146a-5p, miR-181a-5p, miR-195-5p, miR-221-3p, miR-342-3p, miR-499a-5p and miR-574-3p. The performance of ROC curve analyses revealed that these individual microRNAs differentiated at 10.0% FPR between children with abnormal and normal values of echocardiographic measurements, with a sensitivity ranging from 11.32% to 22.64% (Figure S6).

**Figure 6:**
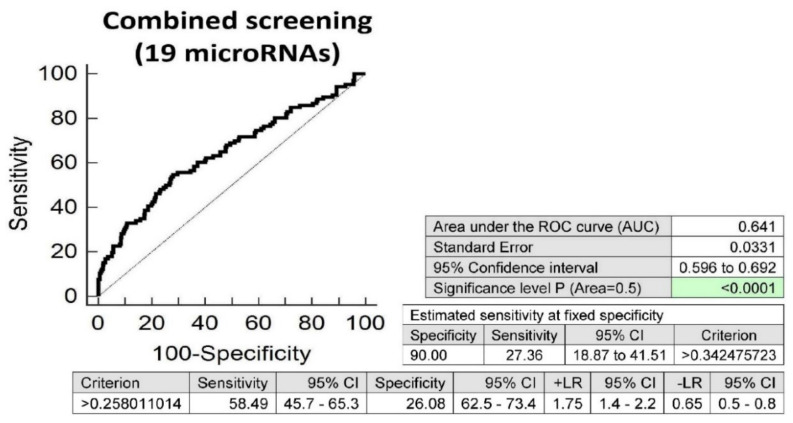
Aberrant microRNA expression profile in children with valve problems or heart defects descending from complicated pregnancies. Combined screening revealed that at 10.0% FPR, 27.36% of children with valve problems or heart defects had a substantially altered microRNA expression profile (miR-1-3p, miR-16-5p, miR-17-5p, miR-20a-5p, mir-20b-5p, miR-21-5p, miR-26a-5p, miR-29a-3p, miR-100-5p, miR-125b-5p, miR-126-3p, miR-143-3p, miR-146a-5p, miR-181a-5p, miR-195-5p, miR-221-3p, miR-342-3p, miR-499a-5p and miR-574-3p).

Screening on the base of a combination of all these 19 microRNAs was superior to the performance of single microRNAs, and showed that at 10.0% FPR, 27.36% children with valve problems or heart defects born from complicated gestation had significantly altered postnatal microRNA expression profile (AUC 0.641, *p* < 0.001, sensitivity 58.49%, specificity 26.08%, cut off >0.258011014) (Figure 6).

## 3. Discussion

At first, we made comparison of microRNA expression profile between children born from complicated and normal gestation, irrespective of the type of pregnancy-related complication (gestational diabetes mellitus, gestational hypertension, preeclampsia, FGR, preterm prelabor rupture of membranes and/or spontaneous preterm birth). Subsequently, we made a comparison of the microRNA expression profile between particular groups of children with respect to actual findings (normal systolic and diastolic blood pressures vs. prehypertension/hypertension; normal BMI vs. overweight/obesity; normal values of echocardiographic measurements vs. the occurrence of valve problems and/or heart defects), regardless of prenatal and perinatal exposure to pregnancy complications (gestational diabetes mellitus, gestational hypertension, preeclampsia, FGR, preterm prelabor rupture of membranes and/or spontaneous preterm birth). In view of the fact that the prenatal and perinatal exposure to any pregnancy-related complication (gestational diabetes mellitus, gestational hypertension, preeclampsia, FGR, preterm prelabor rupture of membranes and/or spontaneous preterm birth) led to substantial alterations of postnatal microRNA expression profile in a proportion of children, we also compared microRNA expression profile between equal groups of children (children born from normal gestation only and children born from complicated gestation only) with respect to actual findings (the occurrence of overweight, obesity, prehypertension, hypertension, valve problems and heart defects).

Substantially altered microRNA expression profile (23 out of 29 tested microRNAs) was identified in a significant proportion of children (57.42% at 10.0% FPR) born from complicated gestation, when compared with children born from normal gestation. All microRNAs (miR-1-3p, miR-16-5p, miR-17-5p, miR-20a-5p, miR-20b-5p, miR-21-5p, miR-23a-3p, miR-26a-5p, miR-29a-3p, miR-100-5p, miR-103a-3p, miR-125b-5p, miR-126-3p, miR-130b-3p, miR-133a-3p, miR-143-3p, miR-146a-5p, miR-181a-5p, miR-195-5p, miR-199a-5p, miR-221-3p, miR-499a-5p and miR-574-3p) were upregulated in the whole peripheral blood of children prenatally or perinatally affected with pregnancy-related complications. We believe that the early life occurrence of alterations in expression of microRNAs involved in the pathogenesis of diabetes mellitus and diverse cardiovascular and cerebrovascular diseases might originate a solid base for predisposition to later onset of diabetes mellitus, cardiovascular and cerebrovascular diseases. For that reason, we consider children exposed to any type of pregnancy-related complication indicated to have altered microRNA expression profile in their peripheral blood leukocytes as the most risky group that should be dispesarized, with the aim of implementing primary prevention measures. Similar findings were reported in children born small for gestational age (SGA), in whom significantly higher circulating levels of miR-16-5p and miR-126-3p were observed in serum samples at the age of 9 years [54].

Parallel to this, we could see that the presence of overweight/obesity and/or valve problems and heart defects had an additional impact on already altered microRNA expression profile in a group of children affected with pregnancy-related complications. A significant proportion of overweight/obese children (42.86% at 10.0% FPR) and children with valve problems and heart defects (27.36% at 10.0% FPR) born from complicated gestation had even higher microRNA expression levels than children from the equal group with actual normal findings. Overweight/obesity intensified upregulation of multiple microRNAs (miR-1-3p, miR-16-5p, miR-17-5p, miR-20a-5p, miR-26a-5p, miR-103a-3p, miR-125b-5p, miR-126-3p, miR-130b-3p, miR-133a-3p, miR-146a-5p, miR-181a-5p, miR-195-5p, miR-199a-5p, miR-221-3p and miR-499a-5p) already present in a group of children previously affected with pregnancy complications. The presence of overweight/obesity induced upregulation of miR-92a-3p and miR-210-3p in children born from complicated gestation. Similarly, abnormal values of echocardiographic measurements also heighten upregulation of some microRNAs, which had already shown altered expression (miR-1-3p, miR-16-5p, miR-17-5p, miR-20a-5p, miR-20b-5p, miR-21-5p, miR-26a-5p, miR-29a-3p, miR-100-5p, miR-125b-5p, miR-126-3p, miR-143-3p, miR-146a-5p, miR-181a-5p, miR-195-5p, miR-221-3p, miR-499a-5p and miR-574-3p) in a group of children prenatally or perinatally affected with pregnancy-related complications. The presence of valve problems and heart defects induced upregulation of miR-342-3p in a group of children born from complicated gestation.

Interestingly, microRNA expression profiles were also able to differentiate between children with abnormal and normal findings (BMI) born from normal gestation. Similarly, as in children born from complicated gestation, the upregulation of multiple microRNAs (miR-16-5p, miR-17-5p, miR-26a-5p, miR-103a-3p, miR-125b-5p, miR-126-3p, miR-130b-3p, miR-146a-5p and miR-181a-5p) was detected in overweight/obese children. In addition, the overweight/obesity induced upregulation of other microRNAs (miR-20b-5p, miR-21-5p, miR-23a-3p, miR-24-3p, miR-29a-3p, miR-92a-3p, miR-100-5p, miR-145-5p, miR-210-3p and miR-574-3p) in children born from normal gestation. Overall, at 10.0% FPR 70.0%, overweight/obese children born from normal gestation demonstrated this abnormal expression profile in their whole peripheral blood. On the other hand, the presence of valve problems or heart defects in children born from normal gestation did not induce any alterations in microRNA gene expression. However, this finding might be influenced by a low number of children with abnormal findings (the occurrence of valve problems or heart defects) in children born from normal gestation.

Differentiation between children with abnormal and normal findings (overweight/obesity, the occurrence of valve problems or heart defects) is also feasible, irrespective of a history of the course of maternal gestation. We identified that at 10.0% FPR, a significant proportion of overweight and obese children (40.63%) had a substantially altered microRNA expression profile. A total of 19 out of 29 tested microRNAs (miR-1-3p, miR-16-5p, miR-17-5p, miR-21-5p, miR-23a-3p, miR-24-3p, miR-26a-5p, miR-92a-3p, miR-100-5p, miR-103a-3p, miR-125b-5p, miR-126-3p, miR-130b-3p, miR-133a-3p, miR-146a-5p, miR-181a-5p, miR-210-3p, miR-221-3p and miR-574-3p) showed increased expression in overweight/obese children. MicroRNA expression alterations of less extent (miR-1-3p, miR-16-5p, miR-20a-5p, miR-21-5p, miR-125b-5p, miR-126-3p, miR-146a-5p, miR-195-5p, miR-221-3p and miR-499a-5p) were also detected in a proportion of children (22.13%) with valve problems or heart defects. Nevertheless, from the data resulting from this study, it is apparent, that the evaluation of potential cardiovascular risk based on the presence of aberrant microRNA expression profile in peripheral blood leukocytes is more accurate with the knowledge of a history of the course of maternal gestation, which might significantly contribute to postnatal modifications of microRNA expression profiles in peripheral blood leukocytes.

Our data may be supported by the data of Chen et al. [55], who recently reported that miR-17-5p is, together with miR-27a/b, a key microRNA playing a role in the pathogenesis of childhood obesity via the regulation of the NLK and RRAS2 genes. Nevertheless, this study was performed using different methodological approach, tissue samples were analyzed using microarray methodology. Similarly, our data are compliant with the data of other investigators, who reported that miR-103a-3p is one of microRNAs implicated in appetite and energy balance control affecting overeating during obesity development including children as well [56,57]. Likewise, Marzano et al. [58] observed upregulation of circulating (serum) miR-92a-3p in obese children, regardless of whether they had been born small for gestational age or appropriate for gestational age.

Circulating (plasma) miR-342-3p was reported, until now, to be associated just with endothelial dysfunction in children, not with other cardiovascular risk factors or other abnormal clinical findings, which is in agreement with our data, even though plasma and whole peripheral blood microRNA gene expression profiles may have different patterns [59].

## 4. Materials and Methods

### 4.1. Participants

The study of a prospective design running within the period of 8/2016–10/2020 involved children of Caucasian descent at the age of 3 to 11 years born from normal pregnancies (*n* = 92) and pregnancies complicated with gestational diabetes mellitus (*n* = 118), gestational hypertension (*n* = 53), preeclampsia (*n* = 135), fetal growth restriction (*n* = 35), preterm prelabor rupture of membranes or spontaneous preterm birth (*n* = 70). The data of children born from normal and complicated gestation are presented in Table 1.

Normal gestation was reported as that one without medical, obstetrical, or surgical complications, where healthy infants with the weight > 2500 g were born after 37 weeks of gestation [52,53].

Gestational diabetes mellitus, glucose intolerance during gestation, was diagnosed following the recommendations of the International Association of Diabetes and Pregnancy Study Groups (IADPSG) [53,60].

Gestational hypertension, hypertension (>140/90 mmHg) with no sign of proteinuria with the first onset after 20 weeks of gestation, was diagnosed following the recommendations of American College of Obstetricians and Gynecologists (ACOG) [52,61].

Preeclampsia was characterized as the occurrence of hypertension and proteinuria (>300 mg/24 h) that appeared firstly after 20 weeks of gestation [52,61]. The severity of preeclampsia was assessed following the recommendations of ACOG [52,61].

FGR, the estimated fetal weight (EFW) below the 3rd percentile or below the 10th percentile for the evaluated gestational age, early FGR (before 32 week of gestation), and late FGR (after 32 week of gestation) were diagnosed based on the current recommendations [52,62,63,64].

Preterm birth (PTB) was defined as the delivery before 37 weeks of gestation at the occurrence of regular uterine contractions along with cervical changes. Preterm prelabor rupture of membranes (PPROM) was diagnosed when amniotic fluid leakage preceded the onset of labor by at least 2 h [65,66,67]. The exclusion criteria for preterm birth (PTB or PPROM) included gestational hypertension, preeclampsia, gestational diabetes mellitus, significant vaginal bleeding and signs of fetal growth restriction.

More details on the definition of individual pregnancy complications are also available in Supplementary Materials.

Children with inborn defects, chromosomal abnormalities and children born from gestation with other complications were excluded from the study.

Informed consent was gained from all study participants. Two ethics committees (with headquarters in the Institute for the Care of the Mother and Child, and the Third Faculty of Medicine, Charles University) granted the approval with the study (grant no. AZV 16-27761A, long-term monitoring of complex cardiovascular profile in the mother, fetus and offspring descending from pregnancy-related complications, dates of approval: 27.3.2014 and 28.5.2015). All procedures were in agreement with the Helsinki Declaration of 1975, as revised in 2000.

### 4.2. Blood Pressure and Echocardiography Measurements, and Body Mass Index Assessment

Standardized blood pressure (BP) and echocardiography measurements and BMI assessment were performed as previously described [52,53,68]. More details are also available in Supplementary Materials.

### 4.3. Processing of Samples, Reverse Transcription, and Relative Quantification of microRNAs

Processing of samples, reverse transcription and relative quantification of microRNAs were performed as previously described [52,53,69,70]. More details are also available in Supplementary Materials.

### 4.4. Statistical Analysis

Statistical analyses (the Shapiro–Wilk test, Mann–Whitney test (M-W), receivers operating characteristic (ROC) curves, and logistic regression combined with ROC curve analysis) were performed as previously described [52,53,71]. The box plots of log-normalized gene expression values (RT-qPCR expression, log_10_ 2^−ΔΔCt^) for particular microRNAs are presented. More details concerning statistical analyses and graphical processing are also available in Supplementary Materials.

## 5. Conclusions

In conclusion, representative microRNA expression profiles for diabetes mellitus, cardiovascular and cerebrovascular diseases may also be detected in peripheral blood leukocytes of children born from complicated gestation (gestational diabetes mellitus, gestational hypertension, preeclampsia, FGR, preterm prelabor rupture of membranes and/or spontaneous preterm birth). This observation implies that pregnancy-related complications of the mother, regardless of its type, may contribute to the predisposition of affected children to later onset of diabetes mellitus, cardiovascular and cerebrovascular diseases. The presence of overweight/obesity and/or valve problems and heart defects have an additional impact on already altered microRNA expression profile in a group of children previously exposed to pregnancy-related complications. MicroRNA expression profiles are also able to differentiate between children with abnormal and normal findings (BMI) born from normal gestation. Consecutive studies are required to confirm the findings of this study.

## 6. Patents

National Patent n. 308102, Industrial Property Office, Czech Republic. PCT/CZ2019/050050, Industrial Property Office, Czech Republic.

## Figures and Tables

**Table 1:** Characteristics of cases and controls.

	NP (*n* = 92)	PE (*n* = 135)	FGR (*n* = 35)	GH (*n* = 53)	GDM (*n* = 118)	PTB (*n* = 33)	PPROM (*n* = 37)	*p*-Value ^1^	*p*-Value ^2^	*p*-Value ^3^	*p*-Value ^4^	*p*-Value ^5^	*p*-Value ^6^
At follow-up													
Age (years)	5 (3–11)	5 (3–11)	4 (3–10)	5 (3–10)	5 (3–10)	5 (3–8)	5 (3–9)	1.000	0.302	1.000	1.000	1.000	1.000
Height (cm)	116.5 (98–153)	114.0 (97–155)	106.0 (93–152)	112.0 (96–159.5)	113.75 (98–153)	115.0 (101–138)	113.5 (102–145)	1.000	<0.001	1.000	1.000	1.000	1.000
Weight (kg)	21.25 14–40.8)	19.25 (11.85–54.9)	16.00 (12–37)	19.70 (14–47.5)	19.50 (14.4–47.1)	19.70 (14.1–29.6)	20.20 (14.7–47.9)	0.331	<0.001	1.000	1.000	1.000	1.000
BMI (kg/m^2^)	15.54 (13.22–20)	14.90 (12.34–22.81)	14.24 (12.7–19.24)	15.42 (13.42–19.7)	15.32 (12.97–20.08)	14.75 (12.09–19.89)	15.86 (13.23–26.28)	0.399	<0.001	1.000	1.000	0.461	1.000
Systolic BP (mmHg)	100.5 (84–123)	99 (84–132)	97 (82–123)	99 (80–129)	100 (82–125)	100 (87–118)	102 (88–125)	1.000	0.812	1.000	1.000	1.000	1.000
Diastolic BP (mmHg)	61 (38–81)	61 (41–88)	60 (42–73)	62 (49–83)	61 (64–122)	61 (43–72)	64 (48–76)	1.000	1.000	1.000	1.000	1.000	1.000
Heart rate (n/min)	90 (51–120)	92 (64–117)	95.5 (62–112)	94 (65–129)	97 (64–122)	95 (76–120)	96 (74–114)	1.000	1.000	1.000	0.002	0.331	0.551
Abnormal echocardiographic findings													
Yes	17 (18.48%)	18 (13.33%)	9 (25.71%)	12 (22.64%)	42 (35.59%)	10 (30.30%)	15 (40.54%)	0.292	0.367	0.546	0.006	0.157	0.009
No	75 (81.52%)	117 (86.66%)	26 (74.29%)	41 (77.36%)	76 (64.41%)	23 (69.70%)	22 (59.46%)						
During gestation													
Maternal age at delivery (years)	32 (20–46)	32 (21–44)	32 (22–41)	32 (27–51)	34 (27–45)	32 (20–39)	32 (22–42)	1.000	1.000	1.000	0.569	1.000	1.000
GA at delivery (weeks)	39.86 (37.71–41.86)	35.86 (26–41.72)	35.14 (28–41)	38.68 (33.43–41.28)	39.57 (37–41.18)	31.0 (24–36.43)	33.43 (24.71–35.86)	<0.001	<0.001	0.002	1.000	<0.001	<0.001
Mode of delivery													
Vaginal	81 (88.04%)	15 (11.11%)	6 (17.14%)	24 (45.28%)	75 (63.56%)	23 (69.70%)	17 (45.95%)	<0.001	<0.001	<0.001	<0.001	0.016	<0.001
CS	11 (11.96%)	120 (88.88%)	29 (82.86%)	29 (54.72%)	43 (36.44%)	10 (30.30%)	20 (54.05%)						
Fetal birth weight (g)	3410 (2530–4450)	2360 (660–4490)	1630 (650–3010)	3150 (1940–4310)	3485 (2700–4400)	1570 (542–2820)	2100 (600–2710)	<0.001	<0.001	0.318	1.000	<0.001	<0.001
Fetal sex													
Boy	47 (51.09%)	57 (42.22%)	18 (51.43%)	26 (49.06%)	74 (62.71%)	20 (60.60%)	15 (40.54%)	0.188	0.973	0.814	0.091	0.347	0.278
Girl	45 (48.91%)	78 (57.77%)	17 (48.57%)	27 (50.94%)	44 (37.29%)	13 (39.40%)	22 (59.46%)						
Primiparity													
Yes	49 (53.26%)	109 (80.74%)	34 (97.14%)	36 (67.92%)	54 (45.76%)	23 (69.70%)	28 (75.68%)	<0.001	<0.001	0.084	0.281	0.101	0.019
No	43 (46.74%)	26 (19.26%)	1 (2.86%)	17 (32.08%)	64 (54.24%)	10 (30.30%)	9 (24.32%)						
Birth order of index pregnancy													
1st	39 (42.39%)	87 (64.44%)	29 (82.86%)	28 (52.83%)	42 (35.59%)	14 (42.42%)	17 (45.95%)	0.007	<0.001	0.471	0.179	0.202	0.790
2nd	34 (36.96%)	28 (20.74%)	2 (5.71%)	13 (24.53%)	45 (38.14%)	9 (27.27%)	12 (32.43%)						
3rd	15 (16.30%)	13 (9.63%)	2 (5.71%)	9 (16.98%)	16 (13.56%)	5 (15.15%)	5 (13.51%)						
4th+	4 (4.35%)	7 (5.19%)	2 (5.71%)	3 (5.66%)	15 (12.71%)	5 (15.15%)	3 (8.11%)						
Infertility treatment													
Yes	3 (3.26%)	34 (25.19%)	9 (25.71%)	7 (13.21%)	16 (13.56%)	4 (12.12%)	6 (16.22%)	<0.001	<0.001	0.023	0.010	0.058	0.009
No	89 (96.74%)	101 (74.81%)	26 (74.29%)	46 (86.79%)	102 (86.44%)	29 (87.88%)	31 (83.78%)						

Data are presented as median (range) for continuous variables and as number (percent) for categorical variables. Significant results are in bold. Continuous variables were compared using the Kruskal–Wallis test. Categorical variables were compared using a chi-square test. *p*-value ^1^: the comparison between normal gestation and PE; *p*-value ^2^: the comparison between normal gestation and FGR; *p*-value ^3^: the comparison between normal gestation and GH; *p*-value ^4^: the comparison between normal gestation and GDM; *p*-value ^5^: the comparison between normal gestation and PTB; *p*-value ^6^: the comparison between normal gestation and PPROM. NP, normal pregnancies; PE, preeclampsia; FGR, fetal growth restriction; GH, gestational hypertension; GDM, gestational diabetes mellitus; PTB, preterm birth; PPROM, preterm prelabor rupture of membranes; BP, blood pressure; CS, Caesarean section; GA, gestational age.

## Supplementary Materials

Supplementary Materials can be found at https://www.mdpi.com/1422-0067/21/21/8413/s1. Figure S1: Aberrant microRNA expression profile in children descending from complicated pregnancies irrespective of the type of pregnancy-related complication (gestational diabetes mellitus, gestational hypertension, preeclampsia, fetal growth restriction, preterm prelabor rupture of membranes, and/or spontaneous preterm birth).; Figure S2: Aberrant microRNA expression profile in overweight/obese children irrespective of the course of gestation of their mothers (normal and complicated pregnancies altogether).; Figure S3: Aberrant microRNA expression profile in children with valve problems or heart defects irrespective of the course of gestation of their mothers (normal and complicated pregnancies altogether).; Figure S4: Aberrant microRNA expression profile in overweight/obese children descending from normal pregnancies.; Figure S5: Aberrant microRNA expression profile in overweight/obese children descending from complicated pregnancies.; Figure S6: Aberrant microRNA expression profile in children with valve problems or heart defects descending from complicated pregnancies.; Table S1: The role of studied microRNAs in the pathogenesis of diabetes mellitus and cardiovascular/cerebrovascular diseases.; Supplementary Material—Methods.

## Abbreviations

GDM	Gestational diabetes mellitus
PE	Preeclampsia
FGR	Fetal growth restriction
GH	Gestational hypertension
AUC	Area under the Curve
ROC	Receive Operating Characteristic
FPR	False Positive Rate
CI	Confidence Interval
LR+	Positive Likelihood Ratio
LR-	Negative Likelihood Ratio
NP	Normal Pregnancies
BP	Blood Pressure
OGTT	Oral glucose tolerance test
IADPSG	International Association of Diabetes and Pregnancy Study Groups
BMI	Body Mass Index
SBP	Systolic Blood Pressure
DBP	Diastolic Blood Pressure
GA	Gestational age
CS	Caesarean Section
